# Qualitative assessment of the intention of Chinese community health workers to implement advance care planning using theory of planned behavior

**DOI:** 10.1186/s12904-021-00885-1

**Published:** 2021-12-10

**Authors:** Bingyu Xing, Guanmian Liang, Jing Zhang, Jinsheng Zhang, Zhizhi Jiang, Qunfang Miao

**Affiliations:** 1 Division of Health Sciences, Hangzhou Normal University, Room 405, Building No.9 in Shenyuan, No.2318 of Yuhangtang Rd, Yuhang District, Hangzhou, 311121 China; 2 Nursing Department, The Cancer Hospital of the University of Chinese Academy of Sciences (Zhejiang Cancer Hospital), Hangzhou, 310022 China; 3 Kaixuan Street Community Health Service Center, Hangzhou, 310000 China

**Keywords:** Community health services, Medical personnel, Advance care planning, Theory of planned behavior

## Abstract

**Background:**

The aging population coupled with progressive medical technology has increased the demand for improved quality of end-of-life in China. However, implementation of an advance care planning (ACP) program in mainland China is still in its infancy owing to the significant influence of filial piety in Chinese culture. Research on implementation of ACP program among community health workers (CHWs) is limited. The current study sought to explore the willingness of CHWs to implement ACP based on the theory of planned behavior (TPB) and provide a reference for promotion of ACP in Chinese communities.

**Methods:**

Phenomenological qualitative study using semi-structured face-to-face interviews. Interviews were audio-recorded. Colaizzi’s method was used for data analysis. The study received ethical approval and all participants provided written consent.

**Results:**

Thirteen CHWs from 3 community health service centers (CHSCs) in Hangzhou, Zhejiang Province, China were interviewed. Through the analysis of the interview content, we determined that most CHWs have a supportive attitude towards the implementation of ACP, the reasons for which are as follows: relieve suffering of patients and respect their medical autonomy; relieve economic and psychological burden on family members; promote development of community palliative care. However, some CHWs believe that the implementation of ACP will lead to doctor-patient disputes and medical risks. CHWs reported that the support of patients and their families, community lawyers, psychosocial professionals, and CHSCs senior managers helped them to implement ACP. In addition, they indicated that the improvement of doctor-patient communication ability, the improvement of community medical environment, the support of government policy, and the training of CHWs were the promoting factors influencing their implementation of ACP. The hindrance factors include insufficient allocation of community health human resources, imperfect ACP legislation in China, and deep-rooted traditional culture.

**Conclusion:**

Findings demonstrated that Chinese CHWs tend to support the implementation of ACP, but their willingness to implement is affected by different factors. CHSCs should actively organize standardized ACP training and comprehensively consider community medical environment, organizational norms, and human resources in implementation of ACP.

**Supplementary Information:**

The online version contains supplementary material available at 10.1186/s12904-021-00885-1.

## Background

Advance care planning (ACP) refers to the process of planning for future healthcare options through discussions between patients, their relatives, and health care professionals or other relevant personnel [1]. It provides a framework for patients to make informed decisions should they lose capacity. ACP respects the autonomy of patients, improves the quality of life of patients at the end of life, and ensures the ultimate care for life [2]. In addition, ACP reduces unnecessary waste of health care resources and minimizes financial and mental burden on families [3].

Although ACP has several benefits, Zhang et al. [4] reported that the public and healthcare professionals in mainland China have low awareness of ACP and underutilize ACP. Therefore, ACP acceptance and implementation is limited in mainland China. The aging population coupled with progressive medical technology has increased the demand for improved quality of end-of-life in China. However, China still lags behind in terms of hospice care and community-based palliative care [5]. The 2015 Economist Intelligence Unit’s Quality of Death Index that covering five categories: palliative care settings, human resources, care affordability, quality of care, and level of community engagement, indicates that China ranks number 71 out of 80 countries and regions in the world [5]. This indicates the urgent need to improve awareness and quality of palliative care services in mainland China. Relevant policies and laws have been established in Europe, America, and other developed countries, as well as Hong Kong and Taiwan to ensure effective implementation of ACP. For instance, the Patient Self-Determination Act (PSDA) in the United States requires that patients be informed of their right to be involved in making decisions regarding the medical care they receive [6]. Moreover, the British Medical Association has approved the Advance Directive [7] and Taiwan passed the “Regulations on Palliative Care” [8]. On the contrary, currently there is no legislation on ACP in mainland China. Although studies report that patients are willing to discuss end-of-life issues and show a positive attitude toward ACP, the response toward ACP implementation remains poor [9]. Family members prefer life support when choosing end-of-life medical treatment to prolong the patient’s life as much as possible owing to the profound influence of traditional filial piety of Chinese culture [10]. In addition, Chinese medical staff choose to actively treat patients rather than offering palliative care [11].

Previous studies have proposed that primary health care institutions are the best starting point for the promotion and implementation of ACP [12]. The American Institute of Medicine reported that hospitals may not meet the needs of patients at the end of their lives, and the ACP made by patients may be unbinding [13]. Reports on sustainability, medical costs, and social promotion, indicate that patient-centered care and family-oriented community health service are critical in development of ACP [13].

Social workers are playing key roles in publicizing, communicating, and assisting the community to understand ACP and related topics in western countries [14]. However, social work professional development in mainland China is immature and the role of ACP is mainly undertaken by community health workers (CHWs). CHW play key roles in providing primary care-based end-of-life care and are therefore more effective in promoting ACP. A study by Kastbom et al. [15] reported that clear medical records and skilled end-of-life communication by health caregivers are important factors that affect implementation of ACP. Studies on implementation of ACP by CHWs in foreign countries exhibit significant advances. The focus has shifted from discussion of conceptual relations, evaluation tools, the current status of cognitive attitudes, and influencing factors to a series of studies on implementation of ACP intervention by CHWs owing to a guarantee and support of laws, policies, and availability of funds [16–19]. ACP studies from mainland China are mainly from hospitals [20, 21], therefore, evidence of ACP implementation by CHWs is limited. CHWs are primary caregivers thus they have close communication and frequent contact with the community members and therefore they are more effective in promoting ACP implementation [22]. In addition, studies on ACP in China are mainly quantitative studies and reviews and only a few qualitative studies apply a complete theoretical framework to comprehensively explore factors that affect ACP implementation by CHWs [23].

The theory of planned behavior (TPB) is one of the most basic and influential theories in the field of human behavioral intention research [24]. The theory postulates that people’s behavior depends on behavioral intentions and behavioral intentions are affected by behavioral attitudes (subjective evaluation of behavior), subjective norms (the degree of influence of significant others on behavior), and perceptual behavior control (facilitating, hindering factors, and self-efficacy of the behavior involved). Notably, these factors are affected by the corresponding behavioral beliefs, normative beliefs, and control beliefs [24]. Several studies have been conducted on ACP to explore the scientific nature and practicability of TPB theory [25, 26].

TPB theoretical framework and qualitative interview technique were used in the current study to explore the factors that affect implementation of ACP by CHWs in the cultural context of mainland China. The findings of this study provide a reference for effective implementation of ACP by community health service centers (CHSCs).

## Methods

### Research design & data collection

This was a phenomenological qualitative study [27], and used individual interviews to explore the subjective perceptions of CHWs on values related to ACP in the context of deep-rooted traditional Chinese culture, and their willingness to implement ACP on patients. This is in line with the previous literature using phenomenological research methods to conduct qualitative research on ACP [28]. The COnsolidated criteria for REporting Qualitative research (COREQ) Checklist [29] was used as underlying structure of this article (see Additional file 1).

Interviews were conducted by BYX and JZ, using an interview guide. The interview guide was designed according to behavioral beliefs, normative beliefs, and control beliefs derived from the TPB. The interview guide was formed after consulting three experts. The professional titles of the 3 experts were associate professor, associate professor, and deputy chief physician. Their research fields were hospice care, medical psychology, and community management, with their working experience being 28, 10, and 18 years according. Before the consultation, BYX first contacted the experts by telephone or interview to explain the purpose of the consultation. After obtaining their consent, BYX invited the three experts and members of the research group to fully discuss and modify the interview outline in the school office. Two main changes were made: (1) “will you initiate ACP discussions at the suggestion of others?” After expert consultation and discussion, this question was considered to be closed and the answer can only be “yes” or “no”. It was thus modified to “Whose advice do you follow regarding initiation of ACP discussions?” (2) “What do you think are the advantages and disadvantages of ACP?” After consultation and discussion by experts, it was agreed that the term “community” should be included, because the research object was community health care workers, and the ultimate purpose of the study was to provide reference data to help community health service centers to effectively carry out ACP. After accepting experts’ advice, it was modified to “What are the advantages and disadvantages of ACP in community?” The interview guidelines after consulting experts are again determined according to the pre interview with two qualified participants (see Additional file 2).

Interviews were conducted at the CHSCs studio to ensure a comfortable and quiet environment for the conversation. Each interview lasted between 30 and 40 min. The recordings were transcribed into text data after 24 h of each interview and reviewed by another researcher. Due to the novelty of the ACP concept, most people in mainland China do not know it [30]. Therefore, before the interview, the researcher distributed the ACP leaflet prepared by the research group to help the interviewee understand the concept and content of ACP (see Additional file 3). In addition, the content of ACP leaflet was reviewed by an expert involved in the hospice care and presented in objective and scientific language as much as possible. The ACP leaflet has no subjective influence on the willingness and influencing factors of CHWs to implement ACP. From the beginning, interviewees were informed that there is no right or wrong answer. During the process of browsing the ACP publicity page, interviewees were asked to avoid imposing their own understanding and judgment on other parties. Both BYX and JZ have experience in qualitative interviews and explained the definition of ACP to interviewees in objective language.

### Participants

In order to answer the research question purposive sampling was used [31]. First, one of the co-authors (Miao) reached out to the manager of the local CHSCs and obtained the manager’s consent to allow us to interview CHWs. BYX and JZ obtained the contact information of CHWs through the managers of CHSCs and sent an invitation letter to participate in the research. Inclusion criteria included individuals who provided community health service for more than a year and volunteered to participate in the study. CHWs interested in participating contacted BYX and JZ to arrange an interview. Participants had no previous knowledge about the study or the interviewer. The sample size is based on the repeated occurrence of data reaching saturation [32].

### Data analysis

Data were analyzed using Nvivo11.0 software. Since ACP is not widely known in mainland China, this study used the Colaizzi’s method to better reflect the neutral and non-judgements of researchers [33]. Colaizzi’s method emphasizes that researchers “suspend” their presuppositions so as to more rigorously explore subjects’ interpretation and understanding of ACP. In previous studies, some scholars [28, 34] used phenomenological research methods to study the quality of ACP, and used Colaizzi’s method to conduct data analysis. Text was analyzed, classified, and summarized in combination with behavioral beliefs, normative beliefs, and control beliefs derived from TPB using Colaizzi’s method. Text analysis involved: (1) careful reading of the transcribed text; (2) identification and extraction of significant statements; (3) coding of recurring ideas from meaningful statements; (4) collection and grouping of encoded views based on the three core variables derived from TPB; (5) writing of a detailed description; (6) summarizing the coding system, identification of similar ideas, and refining of the theme; and (7) going back to the interviewing venue for data verification.

### Ethical considerations

All methods followed the ethical principles for medical research in the Declaration of Helsinki [35]. The study was approved by Ethics Committee of the medical department of Hangzhou Normal University (Reference No. 20190063). Permission was obtained from CHWs before the study commenced. The participants were informed verbally and given written information about the study purpose, confidentiality and voluntariness. Participant names were replaced with a code to ensure anonymity and participants were allowed to stop the interview at any time without giving a reason. Informed consent was given by all participants.

## Findings

### Sample characteristics

In December 2020, we interviewed 13 CHWs, including 7 nurses and 6 doctors, and assigned serial numbers (S1 ~ S13). Demographic characteristics of the participants are presented in Table 1.

**Table 1:** Demographic characteristics of participants

Number	Gender	Age	Department	Education	Post	Professional title	years of working	Years of community work
S1	Female	42	General outpatient	Undergraduate	Nurse	Supervisor nurse	20	5
S2	Female	37	General outpatient	Undergraduate	Nurse	Supervisor nurse	16	3
S3	Female	38	General outpatient	Undergraduate	Nurse	Supervisor nurse	18	6
S4	Female	33	General outpatient	Undergraduate	Nurse	Senior nurse	10	1
S5	Female	35	General outpatient	Undergraduate	Nurse	Senior nurse	15	9
S6	Female	30	Rehabilitation wards	Undergraduate	Nurse	Senior nurse	8	3
S7	Female	45	Rehabilitation wards	Undergraduate	Ward director	Associate chief physician	20	11
S8	Female	50	General outpatient	Junior college	Nurse	Senior nurse	30	30
S9	male	40	General outpatient	Undergraduate	Doctor	Attending doctor	16	16
S10	male	40	Rehabilitation wards	Undergraduate	Doctor	Resident doctor	10	10
S11	Female	42	General outpatient	Undergraduate	Doctor	Associate chief physician	17	13
S12	male	41	General outpatient	Master	Doctor	Associate chief physician	19	19
S13	Female	49	General outpatient	Undergraduate	Doctor	Chief physician	24	15

### Thematic analysis

**Fig. 1:**
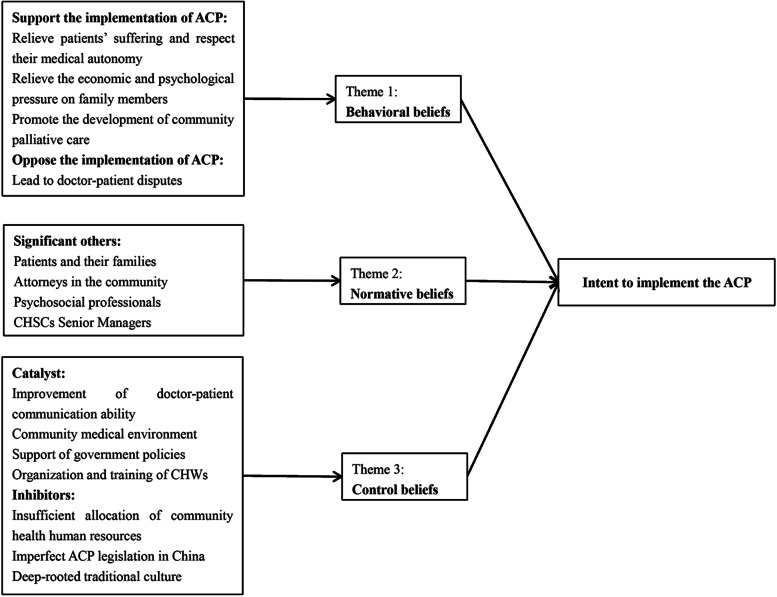
TPB theoretical framework constructed to understand the intention of CHWs to implement ACP

The TPB theoretical model of CHWs’ intention to implement ACP is presented in Fig. 1. Three themes of the behavioral beliefs and normative beliefs of CHWs, and control beliefs of the patient on implementation of ACP were identified during the process of analyzing the content of the interviews. Through the analysis of the interview content, we determined that most CHWs have a supportive attitude towards the implementation of ACP, the reasons for which are as follows: relieve suffering of patients and respect their medical autonomy; relieve economic and psychological burden on family members; promote development of community palliative care. However, some CHWs believe that the implementation of ACP will lead to doctor-patient disputes and medical risks. CHWs reported that the support of patients and their families, community lawyers, psychosocial professionals, and CHSCs senior managers helped them to implement ACP. In addition, they indicated that the improvement of doctor-patient communication ability, the improvement of community medical environment, the support of government policy, and the training of CHWs were the promoting factors influencing their implementation of ACP. The hindrance factors include insufficient allocation of community health human resources, imperfect ACP legislation in China, and deep-rooted traditional culture.

### Theme 1: behavioral beliefs

#### Support the implementation of ACP

Most participants demonstrated positive attitude about ACP. ACP implementation was favored for various benefits as follows.

1. Relieve suffering of patients and respect their medical autonomy.

The two interviewees reported that ACP can alleviate the pain of patients, satisfy their dying wishes and guarantee their medical autonomy, which also reflects the ultimate care for life.

*S3: For example, when some elderly patients in the community are dying, they prefer to stay at home rather than going to a hospital. Therefore, it is reasonable to conduct ACP in the community to meet the dying wish of the elderly.*

*S4: Even if the patient’s life is prolonged, the quality of life is poor and the treatment process is very painful. Therefore, ACP implementation can reduce the degree of pain to some extent.*

(2) Relieve economic and psychological burden on family members.

Chinese society is a family-centered society, and illness is not an individual issue as it affects the entire family. The respondents reported that the long-term treatment process results in a financial burden to the families of patients and causes psychological suffering.

*S3: Some family members may be traumatized by the excruciating medical procedures that their family members undergo before they die such as body tube insertions.*

*S9: In some cases, the rescue efforts may be costly, leading to great economic loss to the family.*

(3) Promote development of community palliative care.

One nurse indicated that ACP can promote palliative care. Palliative care is an important part of ACP, and its tenet of “optimal death” is consistent with ACP’s respect for patients’ autonomy and emphasis on the process of dying with dignity.

*S1: ACP can promote development of palliative care facilities, such as the palliative care facility developed in Shanghai, which we is currently under study. Palliative care is critical for pre-treatment of patients.*

#### Oppose the implementation of ACP

Some participants believed that the implementation of ACP would lead to doctor-patient disputes and medical risks. These negative feelings would thus affect behavioral intention and hinder action. The participants mainly believed that discussing ACP and other death-related topics with patients would cause antipathy of patients. In addition, they reported that the decision-making is inconsistent with that of patients’ family members, thus resulting in doctor-patient disputes.

*S8: ACP involves sober people. If you discuss with patients what to do on the deathbed, they will be exasperated and think you have cursed them, which might lead to an unnecessary medical problem.*

*S10: If the self-consciousness of the patient is unclear, he cannot make a second choice. If the family members disagree with the doctor this may lead to a low degree of trust between the doctor and patient. A lawsuit will be filed if a medical malpractice occurs.*

*S12: Medical risks will occur if the patient chooses not to actively participate in treatment that might lead to disputes between the patient’s family members and medical workers.*

### Theme 2: normative beliefs

#### Support for patients and their families

ACP is implemented mainly for patients. Most participants indicated that the attitude of patients and their families played a decisive role in implementation of ACP by medical caregivers.

*S1: If the patient has a specific idea and can make his own decisions, I will communicate with him on whether ACP should be formulated in advance.*

*S2: Implementation depends on whether patients and their family members agree with ACP or not. I think if they agree, it will be relatively easy to communicate if ACP will be implemented.*

*S10: This kind of promotion depends on the family members’ decision. If no consensus can be reached, there is no way out, even if the elderly firmly agrees to sign the ACP document, when the children do not agree then ACP cannot be implemented.*

#### With the help of a community attorney team

Three respondents emphasized the importance of community lawyers. These participants believed that legal protection plays a key role in implementation of ACP, as they can reduce medical disputes to a certain extent.

*S2:I think it is important not only for the nursing profession, but also for the community which has a legal team.*

*S11: I am just a medical worker, playing a communication role in this, then who will carry out this, who will prove that your documents have legal effect, will there be any disputes, at least there should be community lawyers to help us.*

#### Assessment by psychosocial professionals

Respondents indicated the importance of psychosocial assessments of patients by psychosocial professionals during ACP communication. The participants emphasized that after patients get sick, psychological changes occur in different stages with progression of the disease. Psychological state of patients affects whether they want to discuss sensitive topics like ACP with patients. Therefore, it is necessary for psychiatric professionals to evaluate the psychological state of patients and help them communicate with medical workers.

*S4: You cannot suddenly talk to him about this kind of thing when you don't know his psychological state and the level of acceptance. If his condition changes after discussing the issue then it may be related to changes caused by the discussion.*

*S12: In addition to an assessment of his body, there is also an assessment of his mental state. Sometimes he is not well aware of all aspects of his mental state, so you can't communicate with him the issue on ACP implementation. Therefore, someone with professional psychological knowledge is needed to assess the patient before communicating on ACP implementation.*

#### Attention of CHSCs senior managers

The senior managers of China’s CHSCs include heads of functional departments, heads of clinical divisions, etc [36] In China, senior managers of CHSCs play critical roles in ensuring the success of the implementation of any reform strategies and the effective and efficient service provision in the sector [36]. One respondent pointed out that ACP support by CHSCs senior managers affects their behavioral intentions. Interviewees believe that the guidance of senior health service managers to ACP is important to the implementation of ACP.

*S3: The support from managers is an important aspect. For example, our doctors have medical education departments. Some managers have policies to support us in pre-medical treatment.*

### Theme 2: control beliefs

#### Catalyst

The promoting factors that affect implementation of ACP by CHWs include improvement of doctor-patient communication ability, community medical environment, support of government policies and organization and training of CHWs.

##### Improvement of doctor-patient communication ability

Community medical and nursing staff believe that communication is important in ACP implementation. This includes communication ability and communication opportunity, which can affect the doctor-patient relationship.

*S1: If there are problems in planning ahead, they can be solved through communication. The relationship between the doctor and patient will be strained if communication is not good.*

*S6: Don’t talk directly to the patient at once, but slowly guide him to have the idea of making a medical plan in advance. You can directly state whether you consider being rescued when you get sick in the future. Then I think no one can accept it.*

*S7: This is the importance of timely communication. I cannot inform him of ACP as soon as he is admitted to the hospital. The doctor should choose a suitable time to talk about ACP.*

##### Community medical environment

Four respondents emphasized advantages of implementing ACP in community healthcare settings. In China, large and busy hospitals are mainly involved in first aid and disease treatment, whereas community health service centers mainly carry out hospice and palliative care. In addition, community health service centers provide family-centered medical services and have a harmonious relationship with patients and their families. Therefore, ACP can be discussed effectively under this environment.

*S5: The relationship between patients and the community may be different from doctor-patient relationship in the hospital environment. Many people know and have contact with a family member, thus it may be easier to communicate with them about ACP at the community than in the hospital.*

*S11: Medical and nursing staff in our community may be more familiar with patients, and relatively familiar with patients' conditions. Therefore, it may be easier to promote ACP.*

*S12: With the current community contract services, each doctor may have his fixed patient group, and it will be more easy to promote implementation of ACP.*

##### Support from government policies

The respondents emphasized the importance of government policy support during implementation of ACP. Participants indicated that the government should issue ACP practice guidelines, draw lessons from the practice experience of hospice care, combine China’s national conditions and the current situation of community health service centers, carry out plans according to the practice and application guidelines and constantly improve them during implementation.

*S1: There are a series of rules for palliative care. ACP also needs a series of application standards issued by the government to be promoted in the community before we can implement them.*

*S7: If the government has no policy, it is difficult to carry out the project. It must be led by the government.*

##### Organization and training of CHWs

Community health care workers believe that ACP is a relatively new concept for them, and ACP-related training of medical care groups should be carried out before it is implemented in community health service centers.

*S5: If ACP is promoted here, I think there is need for a formal training for caregivers so that we can communicate with patients after we are familiar with it.*

#### Factors that hinder implementation of ACP

Impeding factors that affect implementation of ACP by CHWs include insufficient allocation of community health human resources, imperfect ACP legislation in China, and deep-rooted traditional culture.

##### Insufficient allocation of human resources for community health

The human resource reserve for community health service centers is still insufficient currently based on the description of the community medical and nursing staff. Participants report that the working time is spent in management of patients’ disease and health, thus there is no time to communicate with patients about ACP.

*S1: We still don't have enough manpower. For instance, I have more than 60 diabetic patients to take care of. Where do I find time to communicate to patients about ACP?*

##### Domestic ACP legislation is not reliable

CHWs emphasized the importance of community lawyers in their normative belief, and in their control belief. In addition, the emphasized the impact of the current lack of ACP legislation in China on implementation of ACP.

*S1: The medical choices the patient makes in advance are invalid unless they have legal support thus we can't carry them out.*

*S4: If we don't have legal support, we won't be allowed to tell patients about these things clinically.*

*S11: Doctors should consider whether ACP is recognized by law or not and whether it violates ethics and certain legal regulations.*

##### Traditional culture is deeply rooted

Moreover, the respondents reported that China’s traditional culture has an important effect on implementation of ACP. Chinese people emphasize the concept of “filial piety comes first”. They are accused of being unfilial if they do not take their family members for treatment. Therefore, filial piety culture limits implementation of ACP.

*S7: Chinese people pay a lot of attention to filial piety. This topic is very sensitive in China and it is taboo to talk about death.*

## Discussion

This qualitative study explored the main beliefs and views of CHWs regarding implementation of ACP in patients in Hangzhou, Zhejiang Province, China. Implementation of ACP was proposed to relieve the patients’ pain associated with disease to some extent and help to alleviate the economic and psychological burden on the family members. In addition, ACP implementation in the community can promote development of community palliative care. The interview indicated that CHWs of the first community hospital were undergoing hospice care training. The participants in this community hospital may have a positive attitude toward ACP owing to the influence of the concept of humanistic care and appropriate care. CHWs had a positive cognition on implementation of ACP and were willing to communicate with patients to formulate ACP. However, some respondents believed that ACP would lead to adverse events, which are attributed to fear of medical disputes caused by improper communication with patients and their families in the process of formulating ACP. This finding was consistent with the results reported in a study by Zhang et al [37]

Implementation of ACP requires the cooperation of a multi-disciplinary team to jointly provide ACP decision support. The most important aspect of ACP is the process of communication between patients and their families and medical professionals. In addition to medical professionals, a multidisciplinary team composed of lawyers, pastors, ACP coordinators, psychological counselors, and social scientists is needed for implementation of ACPs [38]. The ACP Community Guide Program in the United States indicates that ACPs are formulated by multidisciplinary teams [39]. The findings of the present study indicated that community lawyers, psychosocial professionals, and senior managers of CHSCs should be involved in decision-making and support to help implementation of ACP by CHWs in future community health work. In addition, previous studies proposed that, community nurses are the group with the closest contact and most frequent communication with community residents and have more experience in palliative care treatment compared with other medical staff. Nurses offer an important link between patients, families and medical professionals [40]. Nurses can therefore play a key role in disseminating information about ACP and encouraging family discussions about end-of-life care. Hospice care and ACP mainly focus on nursing rather than treatment, thus nurses should be at the heart of the multidisciplinary team implementing ACPs. Therefore, CHSCs should set up a multi-disciplinary team with nurses to provide support from various aspects such as assessing patients’ disease progress, effectively communicating ACP dialogue, determining changes in psychological state, legal provisions and incentive management system, so as to improve professionalism and rationality of ACP decision-making.

Furthermore, most respondents indicated that the attitude of patients and their families towards ACP was the main factor that determined implementation of ACP, which was consistent with the results by Menon et al [41] This indicates that the community should strengthen publicity of ACP knowledge and death education for patients and their families during the process of ACP implementation. Moreover, CHWs should assist patients to communicate with family members about their medical wishes to promote their agreement in the process of ACP formulation.

Control beliefs in the present study were related to several factors, such as doctor-patient communication ability, community medical environment, government policy, organizational training, human resource allocation, ACP legislation and traditional culture. A study conducted in the US explored a community-based ACP communication skills training. The training lasted for 5 weeks and the results showed that trained volunteers can improve the quality and quantity of ACP discussions [39]. A study by Chan et al. [42] reported that trained healthcare professionals are more prepared for ACP in terms of clinical relevance, willingness and confidence, and have a more positive attitude towards ACP implementation. Therefore, the managers of CHSCs should actively conduct relevant training, mainly in the aspect of ACP communication skills, to minimize discomfort during discussion of ACP with patients and alleviate doctor-patient disputes. In addition, ACP implementation is significantly affected by the cultural background, therefore, development of Chinese community ACP should consider patients as the key participants and pay attention to family values, ensure ACP mutual communication between CHWs and patients and their families. In addition, it should ensure that the families understand the patient’s choice, communicate medical decisions to patients and their families, rather than blindly emphasize the autonomy of the patient’s medical condition. In China, CHSCs mainly undertake the professional roles of hospice care and palliative care unlike the busy and institutionalized hospitals which are mainly involved in treatment. Therefore, community health service centers are more suitable to discuss the relatively sensitive ACP topic. Moreover, the community health service centres provide care for patients at home, and good doctor-patient relationship in these facilities makes it easier for patients to discuss their medical decisions with CHWs. These results were consistent with findings reported by Sinclair et al [43] Previous studies report that support of government policies is an important factor in promoting ACP implementation [44]. Development of ACP in communities should be supported by governments at all levels in terms of policies and financial resources [13, 45, 46]. Community ACP in China also required significant support from relevant government departments to improve its standards and implementation process. In the current study, a community contracting policy was suggested to affect ACP implementation. Contracting doctors can help improve doctor-patient relationship and facilitate the link between health care providers and patients and their families, which ultimately promotes dissemination of ACP knowledge. However, the relationship between community policy support and ACP implementation should be verified through further research.

Although participants in the current study reported the benefits of ACP implementation, there are still limitations that affect implementation of ACP in the community. CHWs under the influence of the traditional culture of a happy life believe that ACP involves the topic of the death of patients. Currently, there is no legal practice on the signing and implementation of ACP-related documents in mainland China and its legitimacy is not supported by law. Ma et al. [47] reported that lack of relevant legal support in China is the main factor hindering promotion of ACP. Improvement of the legal guarantee of ACP in the community is important in promoting implementation of ACP by CHWs. Moreover, CHSCs should use ACP brochures, offline lectures, and Internet platforms to strengthen ACP awareness among community residents. In addition, they understand patients’ medical values and attitudes toward death in community health service work, and identify communication opportunities to explain ACP-related content to patients.

### Recommendations

Recommendations for implementing ACP in Chinese CHSCs according to the TPB framework based on the above discussion are presented in Table 2.

**Table 2:** TPB theoretical framework construction for proposing ACP implementation for CHSCs

Belief	Recommendation
Behavioral beliefs	Publicize ACP related information to CHWs, patients and their families. Improve the cognition level of ACP in Mainland China, popularize the concept of “optimal death”, and guide CHWs to form a positive attitude towards ACP.
Normative beliefs	Form multidisciplinary team collaboration to provide ACP decision support.
Control beliefs	1. Take the government as the lead, formulate ACP practice guidelines in accordance with China’s national conditions, and guarantee the legal and economic implementation of ACP. 2. Strengthen training of talent pool of community health service centers to supplement the shortage of human resources. 3. Organize CHWs to attend ACP professional knowledge training and academic seminars. 4. Improve the communication ability of CHWs, and select appropriate ACP communication mode and opportunity.

### Strengths of the study

Previous studies mostly focused on the cognition, attitude and willingness of CHWs to ACP, and did not explore the influence of behavioral beliefs, normative beliefs and control beliefs on behavioral intentions. One of the strengths of this study is to conduct qualitative interviews with CHWs based on a systematic theoretical framework. The purpose is to present CHWs’ true feelings about the ACP concept. At the same time, attention should be paid to eliciting the beliefs behind CHWs’ attitudes and behaviors towards ACP, guiding and helping them to effectively apply ACP to community health service policies.

### Limitations of the study

The study involved participants from only one city in China, and community doctors and nurses in other cities may have different views on implementation intentions of ACP. In addition, the knowledge training experience of medical and nursing staff in different community health service centers is different, thus behavioral intention may vary.

## Conclusion

Prominent beliefs of Chinese CHWs in the implementation of ACP in patients were explored in this study. The results showed that Chinese CHWs have a positive attitude towards ACP, however, the level of their action should be improved. CHSCs should actively organize standardized ACP training and comprehensively consider community health care environment, organizational norms, human resource allocation, law, and other issues, to promote ACP implementation in communities.

## Supplementary Information

**Additional file 1.** The COREQ check list.

**Additional file 2.** Interview guide.

**Additional file 3.** ACP Leaflet.

## Data Availability

Due to the qualitative nature of the study, in order to ensure the data protection and anonymity of respondents, the datasets generated and / or analyzed during the current study are not publicly available. We encourage individual researchers interest in our data to contact us directly (Bingyu Xing, via 993097193@qq.com).

## Abbreviations

ACP: Advance Care Planning
CHWs: Community Health Workers
TPB: Theory of Planned Behavior
CHSCs: Community Health Service Centers
PSDA: Patient Self-Determination Act
COREQ: COnsolidated criteria for REporting Qualitative research
