# Supplementary material for: Qualitative assessment of the intention of Chinese community health workers to implement advance care planning using theory of planned behavior
Source: BMC Palliat Care. 2021 Dec 10;20:187. doi: 10.1186/s12904-021-00885-1 (PMC8662910; doi:10.1186/s12904-021-00885-1)
Supplement: Supplementary file 1 — Additional file 1. The COREQ check list. [file 12904_2021_885_MOESM1_ESM.doc]

**Additional file 1 - The COREQ check list.**

**Table A: Consolidated criteria for reporting qualitative studies (COREQ) checklist.**

| **Domain 1 : Research team and reflexivity** | |
| --- | --- |
| **Personal characteristics** |  |
| 1. Interviewer | Bingyu Xing |
| 2. Credentials | Postgraduate/Master of Science |
| 3. Occupation | Postgraduate/Master of Science |
| 4. Gender | Female |
| 5. Experience & training | Training in qualitative research methods |
| **Relationship with participants** |  |
| 6. Relationship established prior to  study commencement | No |
| 7. Participant knowledge of the  interviewer | Introduce the content and purpose of the research topic to the interviewees before the interview. |
| 8. Interviewer characteristics | The interviewer reported the reasons for the research topic. |
| **Domain 2: Study design** | |
| **Theoretical framework** |  |
| 9. Methodological orientation & theory | TPB* theoretical framework is applied to the content analysis of this study. |
| **Participant selection** |  |
| 10. Sampling | Purpose sampling method |
| 11. Method of approach | Face-to-face interview |
| 12. Sample size | 13 in total |
| 13. Non-participation | Did not arise |
| 14. Setting of data collection | The office of the Community Health Service |
| 15. Presence of non-participants | No |
| 16. Description of sample | This is shown in table 1 of the manuscript |
| **Data collection** |  |
| 17. Interview guide | Topic guide drafted, piloted and revised |
| 18. Repeat interviews | No repeat interviews were conducted |
| 19. Audio/visual recording | Interviews were audio-recorded |
| 20. Field notes | Recorded after interviews |
| 21. Duration | Range 30-40 mins |
| 22. Data saturation | Sampling continued until data saturation |
| 23. Transcripts returned | Return to the interview for confirmation |
| **Domain 3: analysis and findings** | |
| **Data analysis** |  |
| 24. Number of data coders | Outlined in the text, three in total |
| 25. Description of coding tree | A coding tree was not developed, themes were mapped  to the TPB. |
| 26. Derivation of themes | Themes were derived from the data by thematic  content analysis and then mapped to the TPB. |
| 27. Software | NVivo Qualitative Data Analysis Software version 11. |
| 28. Participant checking | This was not conducted |
| **Reporting** |  |
| 29. Quotations presented | Supporting quotations presented |
| 30. Data and findings consistent | Yes |
| 31. Clarity of major themes | A clear presentation of major themes is outlined |
| 32. Clarity of minor themes | Minor themes were discussed |

*TPB =Theory of planned behavior.
