# Supplementary material for: Qualitative assessment of the intention of Chinese community health workers to implement advance care planning using theory of planned behavior
Source: BMC Palliat Care. 2021 Dec 10;20:187. doi: 10.1186/s12904-021-00885-1 (PMC8662910; doi:10.1186/s12904-021-00885-1)
Supplement: Supplementary file 2 — Additional file 2. Interview guide. [file 12904_2021_885_MOESM2_ESM.doc]

**Additional file 2 - Interview guide.**

1. **Behavioral beliefs:**
2. What do you think are the advantages and disadvantages of implementing ACP in the community?
3. **Normative beliefs:**
4. If you want to implement ACP, whom do you think can support/oppose you or influence implementation of ACP in the process?
5. Whose advice would you follow to initiate an ACP discussion?
6. **Control beliefs:**

(1) What factors do you think can promote or hinder initiation of ACP discussions between CHWs and patients?
