# Supplementary figures and images for: Qualitative assessment of the intention of Chinese community health workers to implement advance care planning using theory of planned behavior
Source: BMC Palliat Care. 2021 Dec 10;20:187. doi: 10.1186/s12904-021-00885-1 (PMC8662910; doi:10.1186/s12904-021-00885-1)

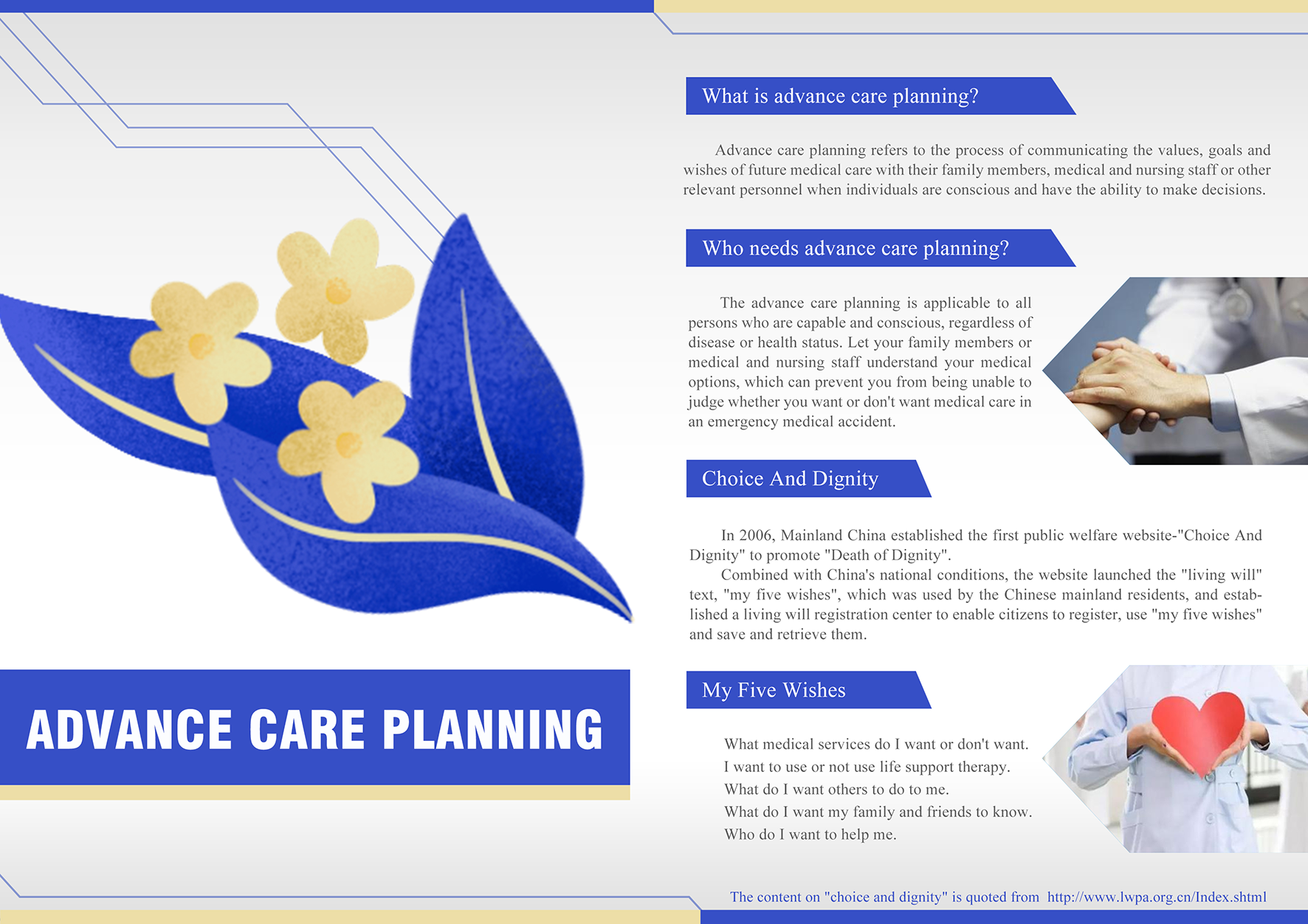

Supplement: Supplementary file 3 — Additional file 3. ACP Leaflet. [file 12904_2021_885_MOESM3_ESM.tif]
